# Abnormal modulation of reward versus punishment learning by a dopamine D2-receptor antagonist in pathological gamblers

**DOI:** 10.1007/s00213-015-3986-y

**Published:** 2015-06-20

**Authors:** Lieneke Katharina Janssen, Guillaume Sescousse, Mahur Melina Hashemi, Monique Harmina Maria Timmer, Niels Peter ter Huurne, Dirk Everdina Maria Geurts, Roshan Cools

**Affiliations:** Donders Institute for Brain, Cognition and Behavior, Radboud University, PO Box 9101, 6500 HB Nijmegen, The Netherlands; Department of Psychiatry, Radboud University Medical Center, PO Box 9101, 6500 HB Nijmegen, The Netherlands; Department of Neurology, Radboud University Medical Center, PO Box 9101, 6500 HB Nijmegen, The Netherlands

**Keywords:** Pathological gambling, Dopamine, Reversal learning, Sulpiride, D2 antagonist

## Abstract

**Rationale:**

Pathological gambling has been associated with dopamine transmission abnormalities, in particular dopamine D2-receptor deficiency, and reversal learning deficits. Moreover, pervasive theoretical accounts suggest a key role for dopamine in reversal learning. However, there is no empirical evidence for a direct link between dopamine, reversal learning and pathological gambling.

**Objective:**

The aim of the present study is to triangulate dopamine, reversal learning, and pathological gambling.

**Methods:**

Here, we assess the hypothesis that pathological gambling is accompanied by dopamine-related problems with learning from reward and punishment by investigating effects of the dopamine D2-receptor antagonist sulpiride (400 mg) on reward- and punishment-based reversal learning in 18 pathological gamblers and 22 healthy controls, using a placebo-controlled, double-blind, counter-balanced design.

**Results:**

In line with previous studies, blockade of D2 receptors with sulpiride impaired reward versus punishment reversal learning in controls. By contrast, sulpiride did not have any outcome-specific effects in gamblers.

**Conclusion:**

These data demonstrate that pathological gambling is associated with a dopamine-related anomaly in reversal learning from reward and punishment.

**Electronic supplementary material:**

The online version of this article (doi:10.1007/s00213-015-3986-y) contains supplementary material, which is available to authorized users.

## Introduction

Pathological gambling is a psychiatric disorder characterized by elevated risk seeking and compulsive gambling behaviour. It can have dramatic consequences including bankruptcy, unemployment, relationship problems and even attempted suicide in up to 24 % of individuals (DeCaria et al. 1996), and its prevalence is estimated between 1 and 2 % in Western countries (Wardle et al. 2010; Welte et al. 2014). In the DSM-5, pathological gambling (renamed *gambling disorder*) is recognized as a behavioural addiction based on similarities with substance addiction in terms of personality traits (impulsivity and compulsivity), clinical symptoms (tolerance, withdrawal, and craving), and associated neurobiological mechanisms (Petry 2007; Potenza 2008, 2013). For example, both substance addiction and pathological gambling have been associated with dopamine transmission abnormalities, in particular dopamine D2-receptor deficiency (Boileau et al. 2013; Clark et al. 2012; Cocker et al. 2012; Comings et al. 1996; Dalley et al. 2007; but also see Joutsa et al. 2012; Linnet et al. 2010). Moreover, pervasive theoretical accounts of addiction suggest a key role for dopamine-dependent abnormalities in reinforcement learning in both substance addiction (Everitt and Robbins 2005; Redish 2004) and pathological gambling (Redish et al. 2007). In these accounts, aberrant reward prediction error signals lead to compulsive over-selection of actions directed at targets of addiction.

Empirical evidence supports the link between dopamine, learning and substance addiction. For example, D2-receptor stimulation has been shown to remediate cognitive impairments in human drug addicts in the context of reversal learning, which reflects the ability to flexibly adapt one’s behavior in response to contingency changes in the environment (Ersche et al. 2011). This concurs with the suggestion that low levels of dopamine D2-receptor availability might predispose to compulsive drug taking (Belin et al. 2008; Dalley et al. 2007) and the claim that reversal learning is a valuable tool for investigating D2-dependent compulsive aspects of pathological reward-seeking behaviours (Izquierdo and Jentsch 2012).

By contrast to substance addiction, there appears to be no empirical evidence for a direct link between dopamine, reversal learning and pathological gambling. Indeed, while research supports links between dopamine and gambling, and between gambling and learning, there is no evidence for dopamine-dependent learning abnormalities in gamblers. Thus, several indices of gambling severity have been associated with lower density of striatal dopamine D2-receptors (Boileau et al. 2013; see also Clark et al. 2012), even though overall group differences have not been reported so far (Linnet et al. 2010). In rodents, D2-receptor agents have been found to influence a behavioural analogue of loss-chasing (Rogers et al. 2013) as well as risk-taking behaviour (St. Onge et al. 2011; Winstanley et al. 2011). Further, gamblers were shown to exhibit diminished ability to update previously learned reward contingencies, as measured with classic instrumental reversal learning tasks (Boog et al. 2014; de Ruiter et al. 2009; Vanes et al. 2014). However, a direct link between dopamine D2-receptor dysfunction, abnormal learning, and pathological gambling is still missing.

The aim of the present study was to triangulate dopamine, gambling and reversal learning by investigating the effects of the dopamine D2-receptor antagonist sulpiride (400 mg) on reversal learning in pathological gamblers. Performance was assessed using a deterministic reversal learning paradigm that enables separate investigation of reward- and punishment prediction learning. This feature of the task is particularly pertinent here, because gamblers have been suggested to be preoccupied with rewards rather than with punishments (Kreussel et al. 2013; Romanczuk-Seiferth et al. 2014). Moreover this paradigm was previously shown to be particularly sensitive to manipulation of dopamine (Cools et al. 2006, 2009; van der Schaaf et al. 2014). Specifically, we have shown that administration of 400 mg of sulpiride to young healthy volunteers altered reward- versus punishment-based reversal learning (van der Schaaf et al. 2014). This finding is in line with a series of other studies (Eisenegger et al. 2014; Frank and O’Reilly 2006; Jocham et al. 2011, 2014) showing effects of D2-receptor blockade in healthy volunteers on reward- versus punishment-based learning and reinforcement-based decisions. As such, this paradigm is a valuable tool to assess whether pathological gambling is accompanied by a dopamine D2-dependent imbalance in learning from reward versus punishment.

This question is particularly relevant in the light of current inconsistencies in the literature regarding the effects of dopaminergic drugs, and specifically dopamine D2-receptor antagonists, in human gamblers. Whereas administration of the D2-receptor antagonist haloperidol has been reported to increase the self-reported desire to gamble in pathological gamblers (Zack and Poulos 2007) and to enhance the impact of reward on betting behaviour (Tremblay et al. 2011), the same drug (although at a lower dose) did not alter subjective, physiological or motivation-to-gamble responses in recreational gamblers in another study (Porchet et al. 2013). So far no study has investigated the effects of D2-receptor antagonism on reward- versus punishment-based learning in human gamblers. Based on previous work (Frank et al. 2004; van der Schaaf et al. 2014), we expected sulpiride to impair reward versus punishment-based learning in healthy controls. In gamblers, we expected impaired punishment versus reward-based learning under placebo. Moreover, we expected that this impairment would be remediated by sulpiride.

## Methods

### Subjects

Twenty-two male pathological gamblers and twenty-two healthy men were included following an in depth structured psychiatric interview administered by a medical doctor (MINI Plus (Sheehan et al. 1998) and the gambling section of the DSM-IV Diagnostic Interview Schedule (Robins et al. 1998)). Two gamblers were excluded from the analyses because of their difficulty understanding the task. An additional two gamblers were excluded from the analyses because of comorbid cannabis dependence within the past 6 months (for details see *Supplementary Materials*). Therefore, the reported results are based on data from 18 gamblers and 22 controls. Supplementary analyses including the two cannabis addicts are reported in the Supplementary Materials and confirmed the effects of primary interest reported in the main text. All subjects provided written informed consent, which was approved by the regional research ethics committee (Commissie Mensgebonden Onderzoek, regio Arnhem-Nijmegen, Registration Number: 2011/204, Date: 14 November 2011), and received compensation for participation.

Pathological gamblers were recruited through advertisement (*n* = 14) and addiction treatment centers (*n* = 4), and reported not to be medicated or in treatment for their pathological gambling at the time of testing. Controls were recruited through advertisement. All gamblers, with the exception of one, qualified as pathological gambler as they met ≥5 DSM-IV-TR criteria for pathological gambling, and were otherwise healthy. One gambler qualified as *problem gambler* as he met only 4 DSM-IV criteria. The severity of gambling symptoms was assessed using the South Oaks Gambling Screen (SOGS; Lesieur and Blume 1987). All gamblers had a minimum SOGS score of 6 (range = 6–18), whereas controls, with the exception of two subjects, had a SOGS score of 0 (range = 0–2).

The two groups were matched for age, net income, body mass index, and verbal IQ as estimated by the Dutch version of the National Adult Reading Test (NLV) (Table 1). Subjects were excluded (from both groups) if they were currently following psychiatric treatment (except cognitive behavioural therapy; *n* = 2); were using more than four alcoholic beverages daily; were using psychotropic medication; had a lifetime history of schizophrenia, bipolar disorder, attention deficit hyperactivity disorder, autism, bulimia or anorexia, anxiety disorder, obsessive compulsive disorder; or had a past 6-month history of major depressive episode. Given the high comorbidity between pathological gambling and other psychiatric disorders (Lorains et al. 2011), gamblers with the following comorbidities were included: lifetime history of dysthymia (*n* = 1); and remitted posttraumatic stress disorder (*n* = 1; remitted > 4 years). Excluding these gamblers from the analyses did not change the results. In addition, three gamblers used cannabis weekly in the past 6 months, but did not meet the DSM criteria for abuse/dependence. Control subjects had no relevant psychiatric history.

**Table 1:** Demographics and self-report measures

	Healthy controls	Pathological gamblers	
n	22	18	
Age	32.2 (2.4)	35.2 (1.9)	*p* = 0.353
Net income	1,715.9 (235.1)	1,750.0 (193.9)	*p* = 0.914
Body Mass Index	23.1 (0.7)	24.1 (0.5)	*p* = 0.280
Education—NART	5.6 (0.2)	5.2 (0.2)	*p* = 0.202
Verbal IQ—NART	105.2 (2.2)	98.7 (2.8)	*p* = 0.072
Digit span—total	15.6 (0.9)	15.1 (0.8)	*p* = 0.691
Number of current smokers	10	12	*p* = 0.180
FTND	0.6 (0.3)	2.9 (0.7)	*p* = 0.002
AUDIT	6.0 (0.8)	7.3 (0.9)	*p* = 0.284
HADS—depression	1.6 (0.5)	4.7 (1.1)	*p* = 0.008
HADS—anxiety	2.6 (0.6)	5.1 (0.8)	*p* = 0.014
BIS-11	57.5 (1.8)	68.3 (2.9)	*p* = 0.002
BIS	17.6 (0.8)	18.4 (0.9)	*p* = 0.502
BAS	38.1 (1.2)	42.3 (1.0)	*p* = 0.013
SOGS	0.2 (0.1)	12.3 (0.9)	*p* < 0.001

If not otherwise stated values represent mean (SEM)

*NART* National Adult Reading Test (Dutch version), *FTND* Fagerstrom Test for Nicotine Dependence, *AUDIT* Alcohol Use Disorders Identification Test, *HADS* Hospital Anxiety and Depression Scale, *BIS-11* Barratt Impulsiveness Scale, *BIS* Behavioural Inhibition System, *BAS* Behavioural Activation System, *SOGS* South Oaks Gambling Screen

Self-report questionnaires were administered to further characterize the subjects (Table 1): the Fagerstrom Test for Nicotine Dependence (FTND; Heatherton et al. 1991), the Alcohol Use Disorders Identification Test (AUDIT; Saunders et al. 1993), the Hospital Anxiety and Depression Scale (HADS; Zigmond and Snaith 1983), the Barratt Impulsiveness Scale (BIS-11; Patton et al. 1995), and the Behavioural Inhibition System/Behavioural Activation System scale (BIS/BAS; Carver and White 1994). Frequent forms of gambling were assessed using item 1 of the SOGS and are expressed in terms of the percentage of gamblers who play the following games at least once a week for money: slot machines (61 %), card games (61 %), casino games (33 %), sports betting (28 %), lotteries (22 %), bowling, pool, golf, darts or alike (5,5 %), stock market (5,5 %).

### Procedure

Subjects visited the lab on two occasions; they were tested once after receiving an oral dose of sulpiride (Dogmatil®, Sanofi-Aventis; 400 mg), and once after a placebo. The order of administration was randomized according to a double-blind, cross-over design. The test sessions were separated by at least one week. Starting time of test sessions was always between 9 and 10 am. Subjects were asked to abstain from recreational drugs 1 week before testing, from alcohol 24 h before testing, and from caffeine and nicotine the morning before testing. The behavioural task was part of a larger protocol and was performed approximately 3.5 h after drug intake, and thus coincided with high plasma concentrations of sulpiride (Mehta et al. 2003).

Background neuropsychological tests (digit span, verbal fluency, number cancellation, and block completion) were administered at the end of the day, 4.25 h after drug intake. Mood, blood pressure and heart rate were measured immediately prior to drug intake, as well as 1 and 4.5 h following drug intake. Subjective mood was measured using the Bond and Lader visual analogue scales (Bond and Lader 1974) and the Positive and Negative Affect Scales (PANAS; Watson et al. 1988).

### Experimental design

**Fig. 1:**
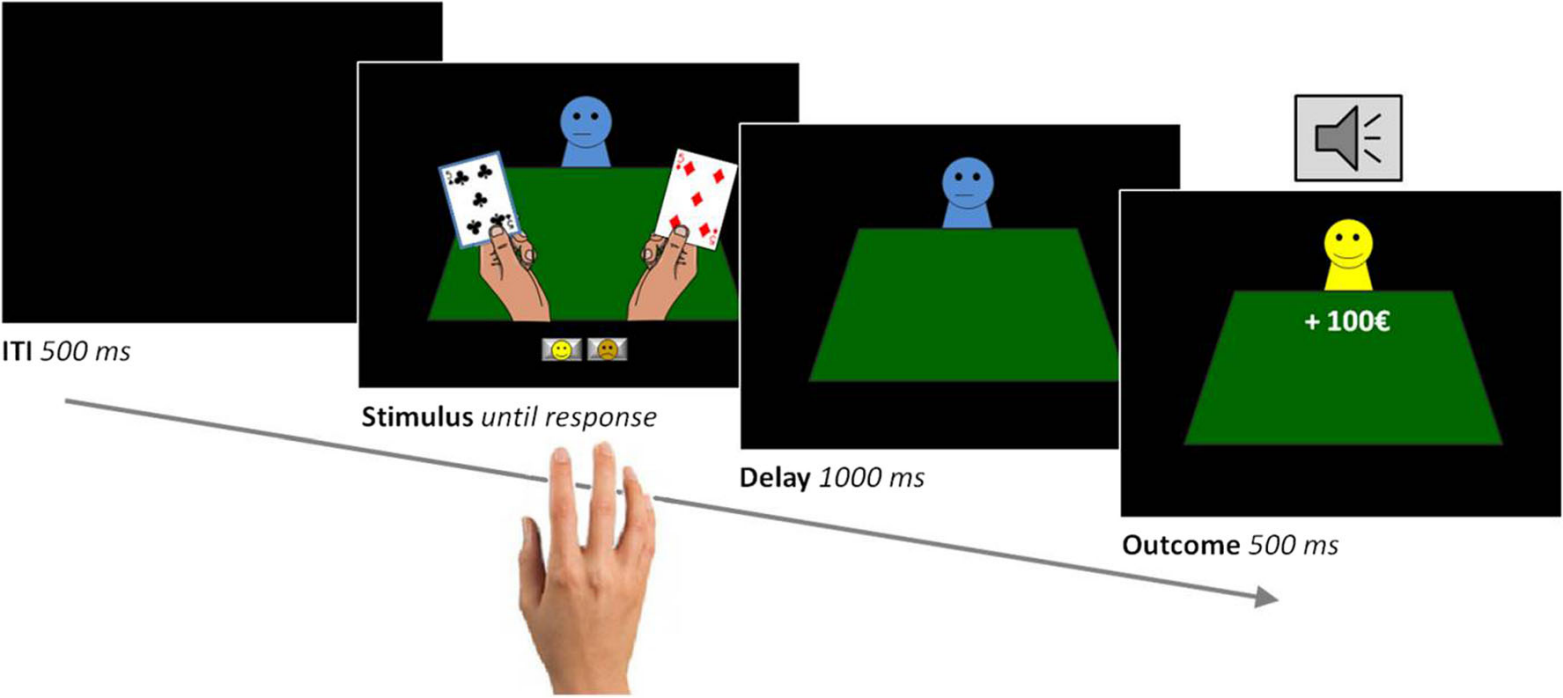
Sample trial of the reversal learning task. On each trial, participants were presented with two gambling cards. One of the cards was selected by computer and highlighted. Participants then had to predict, with a left or right button press, whether the card would be followed by a reward (*a smiling emoticon*, +100€ sign, and a high-pitch tone) or punishment (*a sad emoticon*, −100€ sign, and a low-pitch tone). After a short delay, the outcome was presented. The card-outcome associations were deterministic, and reversed after five to nine correct responses

We employed a deterministic reversal learning task similar to that described elsewhere (Cools et al. 2006; van der Schaaf et al. 2014). The task was programmed with Presentation software (Version 16, Neurobiobehavioral Systems, Inc.). The layout of the task was adjusted to fit the original instructions of a casino setting (see Cools et al. 2006) and to be more intuitive for gamblers. On each trial, subjects were presented with two gambling cards simultaneously (Fig. 1). One of the two cards was associated with upcoming reward, the other one with upcoming punishment. Unlike classic instrumental reversal learning tasks, subjects did not choose between the two stimuli. Instead one card was highlighted, and subjects had to learn to predict the outcome associated with this preselected card by trial-and-error. Responses were made by pressing one of two buttons—one for reward, the other for punishment—with the right index or middle finger (counterbalanced across subjects), and were self-paced. After a 1,000-ms post-response delay the outcome was presented for 500-ms followed by a 500-ms intertrial interval. Note that the outcomes were not contingent on the subjects’ responses, but on the highlighted stimulus; thus, contingencies were Pavlovian rather than instrumental. The stimulus-outcome contingency reversed after five to nine consecutive correct predictions. Subjects performed two blocks, each consisting of two runs of 120 trials (i.e. a total of 480 trials). In one block, reversals were always signaled by unexpected rewards (“reward block”), and in the other block reversals were always signaled by unexpected punishments (“punishment block”). Reward consisted of a smiling emoticon with a “+€100” sign. Punishment consisted of a sad emoticon with a “−€100” sign. The order of blocks was counterbalanced between sessions and across subjects. Error rate on the trials immediately after reversals (i.e. unexpected reward or punishment) indexes the ability to update predictions of reward and punishment, i.e. how well subjects learned from either unexpected reward or unexpected punishment. On these reversal trials, the same stimulus was highlighted as on the previous unexpected outcome trial such that non-outcome-specific requirements for motor switching and prediction updating were matched between reward and punishment conditions. This enabled direct comparison between reward and punishment reversals. Subjects were instructed according to the original procedure by Cools et al. (2006) and were trained extensively before the experiment so that they understood the structure of the task and the Pavlovian, rather than instrumental, nature of the contingencies (for details see *Supplementary Materials*).

### Analyses

Error rates on reversal trials (trials immediately after unexpected outcomes) were arcsine transformed as is appropriate when variance is proportional to the mean (Howell 1997). Error rates on reversal trials were analysed using a mixed ANOVA (SPSS 19, Chicago, IL) with *drug* (placebo vs. sulpiride) and *outcome* (unexpected reward vs. punishment) as within-subject factors and *group* (gamblers vs. controls) as a between-subject factor. In addition, we assessed the total number of reversals obtained throughout the task. Because the stimulus-outcome contingency in the task reversed after five to nine consecutive correct predictions, and the total number of trials was fixed, the number of reversals for the reward and punishment block reflects performance also on the non-reversal trials.

## Results

**Fig. 2:**
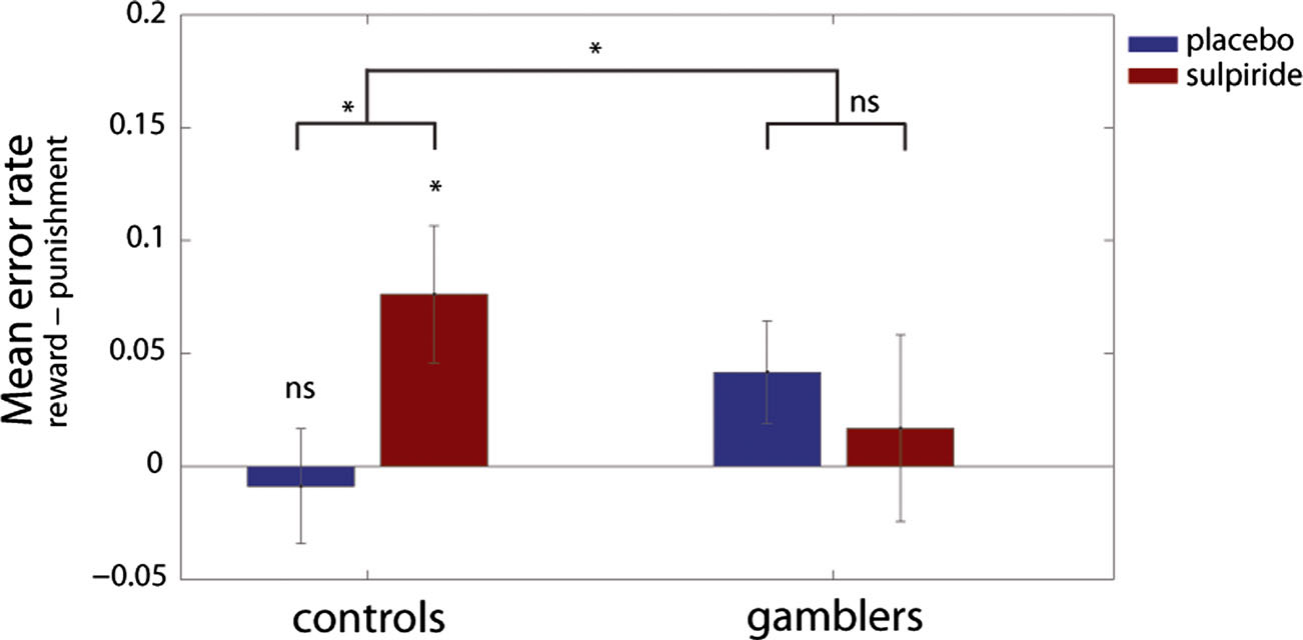
The effect of sulpiride on outcome-specific error rates (i.e., mean error rates on trials following unexpected rewards—mean error rates on trials following unexpected punishment). Sulpiride significantly impairs reward versus punishment learning in controls while not altering the balance between reward and punishment learning in gamblers. *Error bars* represent 1 SEM; *p < 0.05, *ns* denotes not significant

Figure 2 shows that sulpiride altered reward versus punishment reversal learning in controls, while not altering reversal learning in gamblers. This observation was substantiated by an ANOVA of the error rates on reversal trials (Table 2), which revealed a significant interaction of group × drug × outcome (*F*(1,38) = 5.288, *p* = 0.027). When decomposing the three-way interaction effect into two-way interaction effects for each group, we found that this was driven by a drug × outcome interaction in controls (*F*(1,21) = 4.768, *p* = 0.040). By contrast, there was no drug × outcome interaction in gamblers (*F*(1,17) = 1.183, *p* = 0.292). The drug × outcome interaction in controls was due to a significant simple main effect of drug on reward learning (*F*(1,21) = 5.439, *p* = 0.030), not punishment learning (*F*(1,21) = 0.523, *p* = 0.478). Thus, sulpiride induced a shift away from reward learning in controls, while not altering the balance between reward and punishment learning in gamblers. Under placebo there was no group × outcome interaction (*F*(1,38) = 0.976, *p* = 0.329). In addition to the outcome-specific effects of sulpiride on reversal learning, there was also an outcome-nonspecific main effect of drug on error rate (*F*(1,38) = 4.452, *p* = 0.041). This was due to sulpiride impairing performance across groups and outcomes. This raises the question whether the impairment is specific to reversal trials or extends to non-reversal trials. Supplementary analysis including the within-subjects factor *trial type* (reversal, non-reversal reward, and non-reversal punishment trials) revealed a significant group × drug × outcome × trial type interaction (*F*(2,37) = 3.581, *p* = 0.038). When decomposing this interaction into the simple three-way interaction effect for each trial type, we found that this four-way interaction was driven by a group × drug × outcome interaction for reversal trials only. In line with that, there was no significant effect of group, drug or outcome on performance on non-reversal trials as measured by the total number of reversals (Supplementary Fig. Fig. S1).

**Table 2:** Mean error rates on reversal trials

	Healthy controls		Pathological gamblers	
	Placebo	Sulpiride	Placebo	Sulpiride
Reward	0.12 (0.031)	0.17 (0.036)	0.13 (0.031)	0.16 (0.039)
Punishment	0.13 (0.033)	0.10 (0.019)	0.09 (0.023)	0.14 (0.028)

Values represent mean (SEM)

When excluding gamblers with other comorbidities (PTSD and dysthymia; *n* = 2), the effects did not change. The interaction of group × drug × outcome remained highly significant (*F*(1,36) = 7.698, *p* = 0.009). Decomposing the three-way interaction effect into two-way interaction effects for gamblers revealed a marginally significant outcome × drug interaction effect (*F*(1,15) = 3.637, *p* = 0.076) driven by a highly significant impairing effect of drug on punishment learning (*F*(1,15) = 9.370, *p* = 0.008), but not on reward learning (*F*(1,15) = 0.206, *p* = 0.657). Thus in the comorbidity-free pathological gamblers, sulpiride tended to induce a shift away from punishment learning.

The groups did not differ in terms of alcohol use or in terms of the number of smokers, but they did differ significantly in terms of nicotine dependence (FTND), depression and anxiety (HADS), impulsivity (BIS-11), and reward sensitivity (BAS; Table 1). However, there were no correlations between these measures and the drug × outcome interaction effect of interest, suggesting that these between-group differences did not drive the current observations (all |*r*| < 0.35, *p* > 0.155).

Our results are unlikely driven by nonspecific effects of the drug manipulation, because heart rate, blood pressure, mood, and global cognitive function (as measured by background neuropsychological tests) did not significantly differ between the placebo and sulpiride sessions in either group (Supplementary Tables S1-3). Working memory capacity—as a proxy for dopamine synthesis capacity in the striatum (Cools et al. 2008)—was previously shown to predict the effect of sulpiride on learning from reward versus punishment in this paradigm (van der Schaaf et al. 2014). In the current study, working memory capacity did not correlate with the effect of the drug in either group. However, it should be noted that the majority of our sample (independent of group) falls in the low working memory group as reported by van der Schaaf et al. (2014), although the difference in the proportion of high and low working memory capacity subjects between the studies was only marginally significant (cut-off digit span = 17.4; chi(1) = 2.967, *p* = 0.085).

## Discussion

Pathological gambling is thought to implicate dopamine, which is well established to modulate learning from reward versus punishment (Maia and Frank 2011). Like substance addiction, pathological gambling has been hypothesized to be accompanied by dopamine-related impairments in learning (Redish et al. 2007). However, this hypothesis has hitherto never been tested. Here, we establish for the first time a link between pathological gambling, dopamine and learning from reward versus punishment. Specifically, we show that administration of sulpiride, a D2-receptor antagonist, impaired reward- versus punishment-based reversal learning in controls, while not altering reward- versus punishment-based reversal learning in gamblers. However, caution is needed as to the interpretation of the lack of drug effect in gamblers, as supplementary analyses suggest that sulpiride might even have the diametrically opposite effect, i.e. impairing punishment rather than reward-based reversal learning, when only considering gamblers without comorbidities (*n* = 16).

The effect of sulpiride on outcome-specific reversal learning in controls is generally consistent with previous work using the same drug and task in healthy volunteers (van der Schaaf et al. 2014). In this prior study, we showed that the direction of the effect of sulpiride depended on baseline working memory capacity, so that sulpiride impaired reward relative to punishment learning in low working memory participants, whereas it improved reward relative to punishment learning in high working memory participants. The behaviour of our participants is consistent with the impairments observed in low working memory participants. Examination of working memory capacity in our participants showed that the majority of our sample falls in the low working memory group as reported by van der Schaaf et al. (2014). This might reflect the fact that the current sample is more heterogeneous in terms of age and received lower education than the sample of van der Schaaf et al. (2014).

The present results reveal a striking difference in how pathological gamblers and healthy controls respond to the same antipsychotic drug. The differential effect of sulpiride on punishment learning in controls versus non-comorbid gamblers is intriguing and might be relevant in the context of evidence that antipsychotic drugs can impair conditioned avoidance responding (Smith et al. 2004) (although note that our task was not optimized for measuring actual avoidance of punishment). One possibility is that these results reflect an underlying difference in the endogenous dopamine system. In this context, it is interesting to note that pathological gambling has been argued to be accompanied by reduced availability of D2-receptors (Comings and Blum 2000). Some evidence for this hypothesis comes from PET studies, showing that gambling severity and impulsiveness in pathological gamblers correlate with D2/D3-receptor availability (Boileau et al. 2013; Clark et al. 2012). In addition, there is evidence for enhanced drug- and task-induced dopamine release in individuals exhibiting compulsive gambling behaviour (Boileau et al. 2014; Evans et al. 2006; Linnet et al. 2010; O’Sullivan et al. 2011; Steeves et al. 2009).

According to current modeling work of striatal dopamine, D2-receptor blockade might alter reward- versus punishment-based learning and performance by shifting the balance between processing in the D1-mediated GO-pathway and D2-mediated NOGO-pathway of the basal ganglia (Frank 2005; Maia and Frank 2011). In line with the present observation in controls, Pessiglione et al. (2006) found that the D2-receptor antagonist haloperidol impaired reward-learning and attenuated reward prediction error signals in the striatum, suggesting a shift to processing in the D2-mediated NOGO-pathway favouring learning from punishment over learning from reward. Similarly, in a study by Eisenegger et al. (2014) a high dose (800 mg) of the D2-receptor antagonist sulpiride impaired reward-related performance. However, in apparent contrast to those previous findings as well as our current observation in controls, Frank and O’Reilly (2006) found that the D2-receptor antagonist haloperidol improved reward- versus punishment-based learning. In addition, a low dose (200 mg) of the D2-antagonist amisulpride has been found to improve reward- versus punishment-based learning and enhance reward prediction error signal in the striatum (Jocham et al. 2011), suggesting a shift to processing in the D1-mediated GO-pathway. Note however that at a higher dose (400 mg), amisulpride impaired both reward and punishment learning (Jocham et al. 2014). These seemingly paradoxical findings may be explained by the use of different doses, which may in turn lead to differential action of D2-receptor antagonists on pre- vs. postsynaptic receptors in different studies (Frank and O’Reilly 2006). Reward-related improvements with D2-receptor antagonists are generally attributed to action at self-regulatory presynaptic receptors (enhancing dopamine in the synapse), whereas reward-related impairments with D2-receptor antagonists are generally associated with action at postsynaptic receptors. In our study, sulpiride might have shifted the balance away from reward learning in controls, consistent with postsynaptic action, but not in gamblers, suggesting a reduction in postsynaptic action of sulpiride in gamblers versus controls. In fact, when assessing a comorbidity-free group of gamblers, sulpiride tended to actually impair punishment- rather than reward-based learning, raising the possibility that sulpiride might have acted pre- rather than postsynaptically. Preferential sensitivity of pre- versus postsynaptic D2-receptors might make particular sense when synaptic dopamine levels are supra-optimal, e.g. through enhanced dopamine release (Boileau et al. 2014). We emphasize that this hypothesis about the specific mechanism underlying our effect in pathological gamblers remains speculative. One reason is that dopamine D2-receptor antagonists like sulpiride seem to have dose-dependent effects on pre- vs postsynaptic striatal D2-receptors (Frank and O’Reilly 2006). Our dose of 400 mg has been shown to occupy ∼30 % of striatal postsynaptic D2-receptors (Mehta et al. 2008). However, we have no way of quantifying the exact occupancy of pre- and postsynaptic D2-receptors in this study. The pre- versus postsynaptic nature of these D2-receptor effects might be disentangled in future work, for example by administering a higher dose of sulpiride, or by exploiting common polymorphisms in the dopamine receptor D2 gene that are thought to affect the balance between pre- and postsynaptic action (Frank et al. 2007).

One might have expected a baseline difference in reward- and punishment-based learning between the groups. Indeed previous studies have reported slowed learning from punishment on an instrumental reversal learning task (de Ruiter et al. 2009; Vanes et al. 2014) and possibly increased preoccupation with rewards rather than punishments (Kreussel et al. 2013) in pathological gamblers versus controls. Surprisingly, in our study, gamblers and controls learned equally well from unexpected rewards and unexpected punishments under placebo. We are puzzled about this, and hypothesize that this might reflect compensatory mechanisms in the dopamine system, related, e.g. to upregulation of dopamine synthesis capacity, dopamine release, or postsynaptic dopamine receptor sensitivity. This generally concurs with the view that underlying pathology might not surface as impairment under baseline conditions, but only when probing the system, for example by using a pharmacological challenge (Verdejo-García et al. 2008). This also corresponds with the finding that gambling-related abnormalities in baseline D2-receptor availability (Boileau et al. 2013; Clark et al. 2012; Joutsa et al. 2012) are more subtle than these in drug- or task-induced dopamine release (Boileau et al. 2014; Linnet et al. 2010).

In short, we found that blockade of D2-receptors with sulpiride impaired reward versus punishment learning in controls, but not in gamblers. By contrast, in comorbidity-free gamblers, sulpiride impaired punishment, but not reward learning. This strongly suggests that pathological gambling is associated with a dopamine D2-receptor-related anomaly in learning from reward and punishment. Future neurochemical work, using PET or genetics is required to address the exact neurochemical mechanisms of this anomaly.

## Electronic supplementary material

ESM 1 (DOCX 42 kb)

Fig. S1 Mean number of reversals per group (controls, gamblers), drug (placebo, sulpiride), and outcome (unexpected reward, unexpected punishment). There were no significant differences in the number of reversals between groups, drugs, or outcomes. (GIF 107 kb)

High-resolution image (TIFF 1379 kb)
