# Supplementary material for: Abnormal modulation of reward versus punishment learning by a dopamine D2-receptor antagonist in pathological gamblers
Source: Psychopharmacology (Berl). 2015 Jun 20;232(18):3345–53. doi: 10.1007/s00213-015-3986-y (PMC4537492; doi:10.1007/s00213-015-3986-y)
Supplement: Supplementary file 1 — (DOCX 42 kb) [file 213_2015_3986_MOESM1_ESM.docx]

**Supplementary Materials**

**Methods**

***Task instruction and practice***

Subjects were instructed according to the original procedure by Cools et al. ([2006](#_ENREF_11)) and were trained extensively so that they understood the structure of the task and the Pavlovian, instead of instrumental nature of the contingencies; they performed a practice run at the time of intake, and another one before the start of the experiment on each test day. On the intake, the practice run consisted of two stages, one initial acquisition stage and one reversal stage. Following attainment of an initial learning criterion of 20 correct trials, the practice run proceeded to the reversal stage. The practice run was terminated if the subject reached a learning criterion of 20 correct trials in the reversal stage or if a maximum of 80 trials was performed. On the test days, the practice run consisted of 40 trials of the actual task, in which the stimulus-outcome contingency reversed after 5 to 9 consecutive correct predictions. Subjects were required to perform above chance-level to proceed to the actual task. Subjects who did not perform above chance-level were instructed again and did the practice run once more (n_controls_=4, n_gamblers_=2). No more than two practice sessions were required for any of the subjects.

***Exclusion of subjects***

Two subjects were excluded because of difficulty understanding the task. Both subjects passed the learning criterion of the practice run after thorough instructions, but on several runs of the actual task their error rates were three standard deviations above the group mean.

An additional two gamblers were excluded from the analyses because of comorbid cannabis dependence within the past six months. These subjects were initially included because we could not predict how the recruitment of a comorbidity-free sample of gamblers would go. However, given the association between substance dependence, dopamine and reversal learning, our results could be biased by including subjects with comorbid cannabis dependence (Belin et al., 2008; Comings and Blum, 2000; Dalley et al., 2007; Ersche et al., 2011). Since we were successful in including a sufficiently large sample of gamblers without comorbidities, we finally decided to exclude gamblers with comorbid cannabis dependence.

**Results**

Including the gamblers with comorbid cannabis-dependence (within the past six months) in the analysis (n=2) confirmed the effects of primary interest reported in the main text. ANOVA of error rates on reversal trials revealed a marginally significant interaction of group x drug x outcome (F(1,40) = 3.408, p = 0.072). When decomposing the three-way interaction effect into two-way interaction effects for each group, we found that this was driven by a drug x outcome interaction in controls (F(1,21) = 4.768, p = 0.040). By contrast, there was no drug x outcome interaction in gamblers (F(1,19) = 0.180, p = 0.677. Indeed there was a strong simple main effect of outcome in the sulpiride condition in controls (F(1,21) = 8.270, p = 0.009) but not in gamblers (F(1,19) = 0.014, p = 0.906).

**References**

Belin, D., Mar, A.C., Dalley, J.W., Robbins, T.W., and Everitt, B.J. (2008). High impulsivity predicts the switch to compulsive cocaine-taking. Science *320*, 1352-1355.

Comings, D.E., and Blum, K. (2000). Reward deficiency syndrome: genetic aspects of behavioral disorders. In Progress in Brain Research (Elsevier).

Dalley, J.W., Fryer, T.D., Brichard, L., Robinson, E.S., Theobald, D.E., Lääne, K., Peña, Y., Murphy, E.R., Shah, Y., and Probst, K. (2007). Nucleus accumbens D2/3 receptors predict trait impulsivity and cocaine reinforcement. Science *315*, 1267-1270.

Ersche, K.D., Roiser, J.P., Abbott, S., Craig, K.J., Müller, U., Suckling, J., Ooi, C., Shabbir, S.S., Clark, L., Sahakian, B.J.*, et al.* (2011). Response perseveration in stimulant dependence Is associated with striatal dysfunction and can be ameliorated by a D2/3 receptor agonist. Biological Psychiatry *70*, 754-762.

**Tables**

Table S1. Physiological measures. ANOVAs for each of the measures showed no main effect of drug, nor any interactions with drug, ruling out a potential non-specific effect of the drug.

|  |  | **Healthy controls** | |  | **Pathological gamblers** | |
| --- | --- | --- | --- | --- | --- | --- |
|  |  | **Placebo** | **Sulpiride** |  | **Placebo** | **Sulpiride** |
| Heart rate | T1 | 70.8 (2.2) | 70.0 (2.3) |  | 66.8 (1.9) | 69.4 (1.7) |
|  | T2 | 61.7 (2.0) | 62.4 (1.3) |  | 59.7 (2.0) | 60.6 (2.1) |
|  | T3 | 64.6 (1.3) | 64.1 (1.7) |  | 65.4 (2.5) | 64.4 (2.5) |
| Systolic blood pressure | T1 | 129.4 (2.4) | 130.2 (2.7) |  | 127.7 (2.7) | 127.4 (3.1) |
|  | T2 | 127.1 (2.1) | 130.6 (2.7) |  | 125.9 (3.0) | 126.3 (2.9) |
|  | T3 | 128.1 (2.5) | 128.0 (3.0) |  | 129.4 (2.9) | 127.1 (2.3) |
| Diastolic blood pressure | T1 | 75.6 (1.8) | 76.6 (1.8) |  | 75.1 (2.0) | 74.8 (2.2) |
|  | T2 | 76.7 (1.6) | 78.9 (2.6) |  | 75.6 (2.1) | 74.2 (1.6) |
|  | T3 | 77.5 (1.6) | 76.4 (1.7) |  | 75.2 (1.9) | 75.1 (1.5) |

*Values represent mean (SEM). T1: time point 1, before drug intake; T2: time point 2, 1.h after drug intake; T3: time point 3, 4.5h after drug intake*

Table S2. Mood ratings, as assessed by the Bond and Lader and PANAS scales. ANOVAs for each of the measures showed no main effect of drug, nor any interactions with drug, ruling out a potential non-specific effect of the drug.

|  |  |  | **Healthy controls** | |  | **Pathological gamblers** | |
| --- | --- | --- | --- | --- | --- | --- | --- |
|  |  |  | **Placebo** | **Sulpiride** |  | **Placebo** | **Sulpiride** |
| Bond and Lader | Alertness | T1 | 78.0 (3.1) | 77.7 (3.2) |  | 78.0 (3.6) | 76.4 (5.2) |
|  |  | T2 | 83.8 (2.7) | 78.4 (3.2) |  | 77.7 (3.8) | 81.8 (3.8) |
|  |  | T3 | 84.3 (2.6) | 84.3 (2.3) |  | 76.0 (5.3) | 77.2 (4.8) |
|  | Calmness | T1 | 76.9 (3.0) | 76.1 (3.3) |  | 72.6 (5.3) | 71.0 (5.4) |
|  |  | T2 | 79.2 (3.5) | 75.3 (3.4) |  | 75.7 (5.3) | 77.1 (4.1) |
|  |  | T3 | 84.5 (2.7) | 84.0 (2.4) |  | 79.1 (4.1) | 81.6 (3.8) |
|  | Contentedness | T1 | 75.5 (3.5) | 80.0 (2.9) |  | 77.1 (4.9) | 77.1 (5.4) |
|  |  | T2 | 80.9 (3.8) | 81.7 (2.6) |  | 77.7 (4.6) | 81.5 (4.7) |
|  |  | T3 | 83.5 (3.2) | 84.3 (2.3) |  | 83.0 (3.5) | 79.6 (4.3) |
| PANAS | Positive affect | T1 | 35.9 (1.5) | 32.6 (1.4) |  | 34.6 (1.9) | 34.6 (2.1) |
|  |  | T2 | 34.1 (1.6) | 33.5 (1.2) |  | 34.3 (2.2) | 35.1 (2.3) |
|  |  | T3 | 34.7 (1.2) | 33.6 (1.1) |  | 34.1 (2.4) | 33.6 (2.5) |
|  | Negative affect | T1 | 12.3 (0.8) | 14.3 (1.8) |  | 16.5 (1.7) | 14.5 (1.2) |
|  |  | T2 | 11.9 (1.0) | 12.1 (0.7) |  | 13.5 (0.8) | 14.4 (1.3) |
|  |  | T3 | 12.5 (0.9) | 12.5 (0.7) |  | 13.0 (0.7) | 12.9 (1.1) |

*Values represent mean (SEM). T1: time point 1, before drug intake; T2: time point 2, 1.h after drug intake; T3: time point 3, 4.5h after drug intake.*

Table S3. Global cognitive function as assessed by classical neuropsychological tests (performed at the end of each test day). ANOVAs for each of the measures showed no main effect of drug, nor any interactions with drug, ruling out a potential non-specific effect of the drug.

|  |  | **Healthy controls** | | **Pathological gamblers** | |
| --- | --- | --- | --- | --- | --- |
|  |  | **Placebo** | **Sulpiride** | **Placebo** | **Sulpiride** |
| **Digit span** | **forward** | 8.3 (0.5) | 8.4 (0.6) | 8.0 (0.5) | 8 (0.5) |
|  | **backward** | 7.3 (0.6) | 7.4 (0.5) | 7.2 (0.4) | 7.0 (0.5) |
|  | **total** | 15.6 (1.0) | 15.8 (0.9) | 15.2 (0.8) | 15.1 (0.8) |
| **Number cancellation** | **time** | 241.1 (10.2) | 245.0 (8.2) | 225.0 (8.9) | 225.2 (9.2) |
|  | **#misses** | 3.5 (0.8) | 3.4 (0.7) | 4.4 (1.3) | 5.8 (2.2) |
| **Block completion** | **Time** | 70.0 (3.6) | 71.6 (4.0) | 75.7 (3.4) | 76.8 (5.3) |
| **Verbal fluency** | **#words** | 38.6 (2.1) | 39.5 (1.8) | 41.3 (2.7) | 40.9 (2.4) |

*Values represent mean (SEM).*
