# Supplementary figures and images for: Abnormal modulation of reward versus punishment learning by a dopamine D2-receptor antagonist in pathological gamblers
Source: Psychopharmacology (Berl). 2015 Jun 20;232(18):3345–53. doi: 10.1007/s00213-015-3986-y (PMC4537492; doi:10.1007/s00213-015-3986-y)

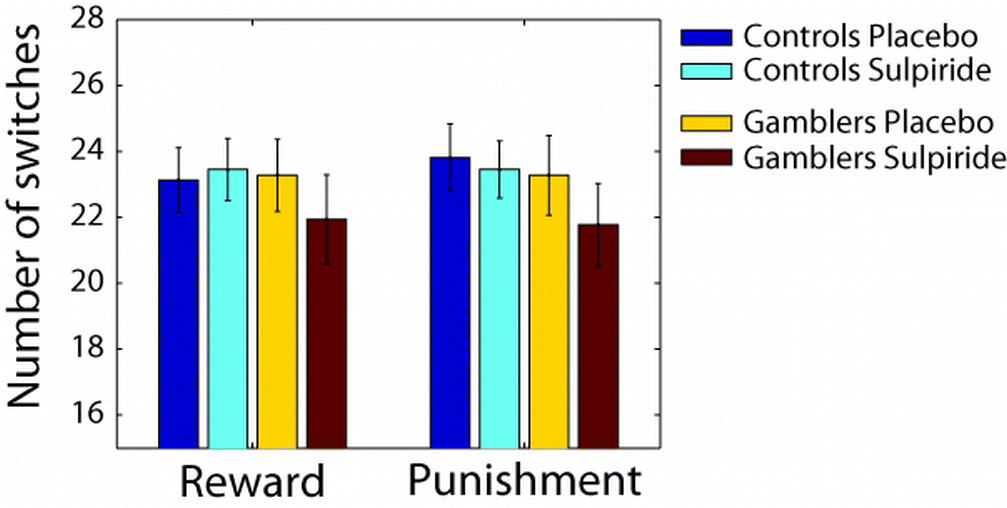

Supplement: Supplementary file 2 — Mean number of reversals per group (controls, gamblers), drug (placebo, sulpiride), and outcome (unexpected reward, unexpected punishment). There were no significant differences in the number of reversals between groups, drugs, or outcomes. (GIF 107 kb) [file 213_2015_3986_Fig3_ESM.gif]

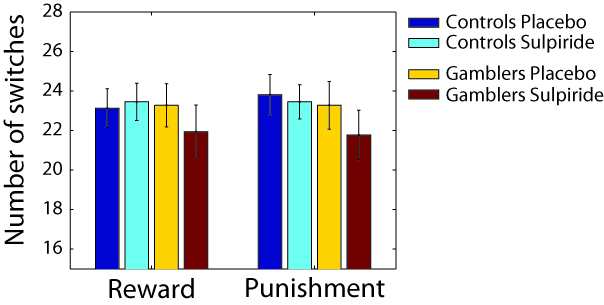

Supplement: Supplementary file 3 — High-resolution image (TIFF 1379 kb) [file 213_2015_3986_MOESM2_ESM.tif]
